# 3-carbamoyl proxyl nitroxide attenuates CCl_4_-induced liver fibrosis in mice through antioxidant-inflammatory regulation of TLR4/NF-κB signaling pathway

**DOI:** 10.1038/s41598-026-46137-1

**Published:** 2026-03-27

**Authors:** Ru Yao, Rong Wang, Yujie Wang, Lu Han, Panpan Chen, Fangbin Liu, Lei Wang, Yongfang Yuan

**Affiliations:** 1 Department of Pharmacy, Shanghai Ninth People’s Hospital, Shanghai JiaoTong University School of Medicine, 280 Mohe Rd, Shanghai, 201999 China; 2 College of Clinical Pharmacy, Shanghai Jiao Tong University School of Medicine, Shanghai, China

**Keywords:** Liver fibrosis, Inflammation, Nitroxide radical, Hepatic stellate cell, NF-κB, Biochemistry, Cell biology, Diseases, Drug discovery, Gastroenterology, Immunology, Medical research, Molecular biology

## Abstract

Liver fibrosis is a dynamic pathological consequence of chronic liver injury, in which persistent oxidative stress and inflammation drive progressive extracellular matrix deposition. Cyclic nitroxide radicals exhibit diverse biological activities, but their effects on liver fibrosis remain unclear. This study systematically evaluates the therapeutic potential of 3-carbamoyl proxyl nitroxide (3-CP) against carbon tetrachloride (CCl₄)-induced liver fibrosis. In vitro, 3-CP inhibited hepatic stellate cell (HSC) activation, migration, and proliferation, and reduced α-smooth muscle actin (α-SMA) and collagen I (COL1) expression. In a BALB/c mouse model of CCl4-induced liver fibrosis, 20 and 40 mg/kg 3-CP reduced the fibrosis area from 13.6 ± 1.0% (model group) to 6.9 ± 0.9% and 5.7 ± 1.3%, respectively, accompanied by decreased serum transaminase levels, restored liver architecture, and diminished collagen deposition. Mechanistic studies revealed that 3-CP modulated the TLR4/NF-κB signaling pathway, downregulating phosphorylated NF-κB p65 (p-p65) and reducing hepatic mRNA levels of pro-inflammatory (IL-1β, IL-6, TNF-α) and pro-fibrotic (TGF-β) cytokines by approximately 35–55%. Supportive in silico analysis suggested potential interactions between 3-CP and key pathway proteins (TLR4, MyD88, IKKβ, p65, IκBα). These findings indicate that 3-CP represents a promising therapeutic candidate that concurrently addresses oxidative damage and inflammatory signaling during liver fibrogenesis.

**Supplementary Information:**

The online version contains supplementary material available at 10.1038/s41598-026-46137-1.

## Introduction

Liver fibrosis is a common pathological process in chronic liver disease and represents an abnormal repair response following chronic liver injury^1,2^. Following liver injury, quiescent hepatic stellate cells (HSCs) undergo activation and transformation into myofibroblasts^3^, and this transformation is intrinsically associated with the synthesis of extracellular matrix (ECM) components, including α-smooth muscle actin (α-SMA) and type I collagen (COL1)^4^. A key driver of this process is the excessive generation of reactive oxygen species (ROS) and the sustained activation of inflammatory pathways^5^. ROS overproduction directly damages hepatocytes and disrupts endothelial integrity, facilitating inflammatory cell infiltration and creating a pro-oxidant, pro-inflammatory microenvironment that activates HSCs^6–8^.

Elevated serum lipopolysaccharide (LPS) during chronic liver injury engages toll-like receptor 4 (TLR4) on hepatocytes, triggering an innate immune response that culminates in nuclear factor-κB (NF-κB) activation and nuclear translocation^9–12^. The signaling pathway, which is highly conserved and strictly regulated, is essential for liver cell survival and hepatic homeostasis. It modulates the expression of inflammatory factors and triggering liver inflammation^13^. Several studies, including our previous work, have shown that the inhibition of NF-κB activity can prevent liver fibrosis in murine models^14–16^. Collectively, an increasing body of evidence has highlighted the significant role of the TLR4/NF-κB signaling pathway in the pathogenesis of liver fibrosis^17^. Despite advances in understanding its pathogenesis^18,19^, no FDA-approved therapies specifically target liver fibrosis^20–22^. Therefore, strategies aimed at reducing oxidative stress and inflammation to regulate NF-κB expression and activity may emerge as effective therapeutic options for the treatment of liver fibrosis^23^.

Nitroxide radicals are a class of stable free radicals that exhibit diverse biological activities like anti-inflammation^24,25^, anti-oxidation^26,27^, and anti-tumor effects^28^. Unlike traditional antioxidants, they protect cells by catalyzing free-radical detoxification and easily crossing cell membranes and the blood-brain barrier^29^. Nitroxide-based drugs (e.g., Tempol) can regulate free radical-induced oxidative stress and inflammation, showing therapeutic potential in multiple disease models^30,31^. Blonder et al. found that tempol-labeled human serum albumin (HSA) reduced liver inflammation and related cell accumulation after injury and ischemia-reperfusion^32^. Notably, 3-carbamoyl proxyl (3-CP), a nitroxide analog, has demonstrated superior efficacy to Tempol in reducing inflammatory cell recruitment and attenuating bleomycin-induced pulmonary fibrosis in preclinical models^33,34^. Furthermore, related nitroxides have shown favorable safety profiles in early-phase clinical trials^35,36^. While these findings are encouraging, the specific molecular mechanisms by which nitroxide radicals exert anti-fibrotic effects in the liver remain largely unexplored. This knowledge gap, together with the favorable pharmacokinetic profile and superior anti-inflammatory activity of 3-CP relative to other nitroxides (e.g., Tempol), prompted us to investigate its therapeutic potential in liver fibrosis.

In this study, we hypothesized that 3-CP has the potential to alleviate liver fibrosis, and verified this in an LPS-induced HSC cell model and a murine model of CCl_4_-induced liver fibrosis. Given the established association between the pathogenesis of liver fibrosis and the TLR4/NF-κB signaling pathway, this study aimed to elucidate the potential mechanism underlying the anti-fibrotic effect of 3-CP via the TLR4/NF-κB signaling pathway, thereby providing a theoretical basis for the utilization of nitroxide radicals in liver fibrosis management.

## Results

### 3-CP inhibited the proliferation, migration and promoted apoptosis of HSCs

**Fig. 1:**
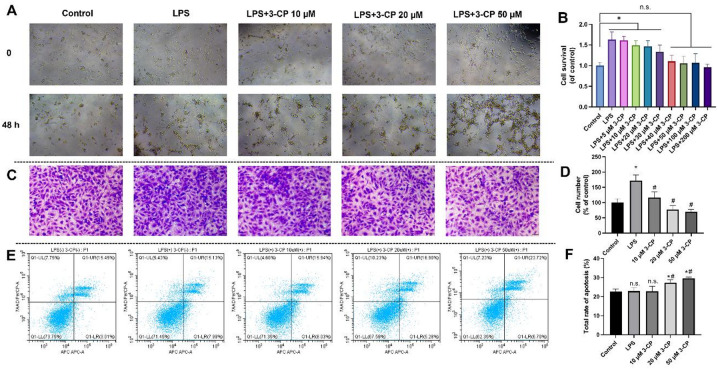
3-CP inhibited the proliferation, migration and promoted apoptosis of LX2. (**A**) Morphology of LX2 cells after coincubation with LPS and 3-CP. (**B**) The effect of 3-CP on LPS-induced LX2 cell proliferation was measured using CCK-8 assay. Data are presented as the mean ± SD, *n* = 4. *p < 0.05, vs. control group; #*p* < 0.05, vs. LPS group. (**C**-**D**) Migration of LX2 cells after co-incubation with LPS and 3-CP was determined using a transwell assay (magnification, ×200). (**E**) Contour diagram of Annexin V-APC/7-AAD flow cytometry of LX2 cells. (**F**) Calculation of apoptotic cells using flow cytometry for each group. Data are presented as mean ± SD, *n* = 3. *p < 0.05, vs. control group; #*p* < 0.05, vs. LPS group.

A CCK-8 assay was performed to assess the effect of 3-CP on LX2 inflammation and a hepatic fibrosis model stimulated by LPS (200 ng/mL), and to determine the optimal concentration of 3-CP. As shown in Fig. 1A-B, CCK-8 results revealed that the proliferation capacity of LX2 cells gradually decreased with increasing the concentration of 3-CP. Notably, no significant cytotoxicity was observed at concentrations up to 50 µM, and the viabilities at 40 µM and 100 µM 3-CP were comparable to the control group, indicating that 3-CP is well-tolerated by HSCs at the selected concentrations. Therefore, 10, 20, and 50 µM 3-CP were selected as the final concentrations to ensure drug effectiveness without affecting cell growth.

Moreover, the cell migration experiments exhibited a trend similar to that of HSC proliferation. During liver fibrosis, activated HSCs contribute to fibrosis through proliferation and migration. As depicted in Fig. 1C, LX2 cells treated with 200 ng/mL lipopolysaccharide (LPS) and varying concentrations of 3-CP (10, 20, and 50 µM) showed a concentration-dependent reduction in chemotaxis compared to cells treated with LPS alone. These findings indicated that 3-CP treatment significantly inhibited HSC migration.

To further validate the effect of 3-CP on the proliferation of HSC, the cell cycle and apoptosis of LX2 cells were evaluated using flow cytometry, apoptotic cells were quantified as the sum of early (Q1-LR) and late (Q1-UR) apoptotic populations. Compared with the control group (LPS (-)/3-CP (-)), there was no significant difference in the apoptosis rate between the LPS-stimulated group and the low concentration 3-CP group (LPS (+)/3-CP (10 µM)). Compared to the LPS-stimulated group, the apoptosis rate of the 3-CP group (20 µM and 50 µM) increased significantly (*p* < 0.05) (Fig. 1E-F). Interestingly, the pro-apoptotic effect appeared to plateau between 20 µM and 50 µM, with no significant difference observed between these two concentrations, suggesting saturation of the apoptotic response at higher doses. In summary, 3-CP inhibits the proliferation and migration of HSCs and promotes their apoptosis.

### 3-CP inhibits ROS content of HSCs

**Fig. 2:**
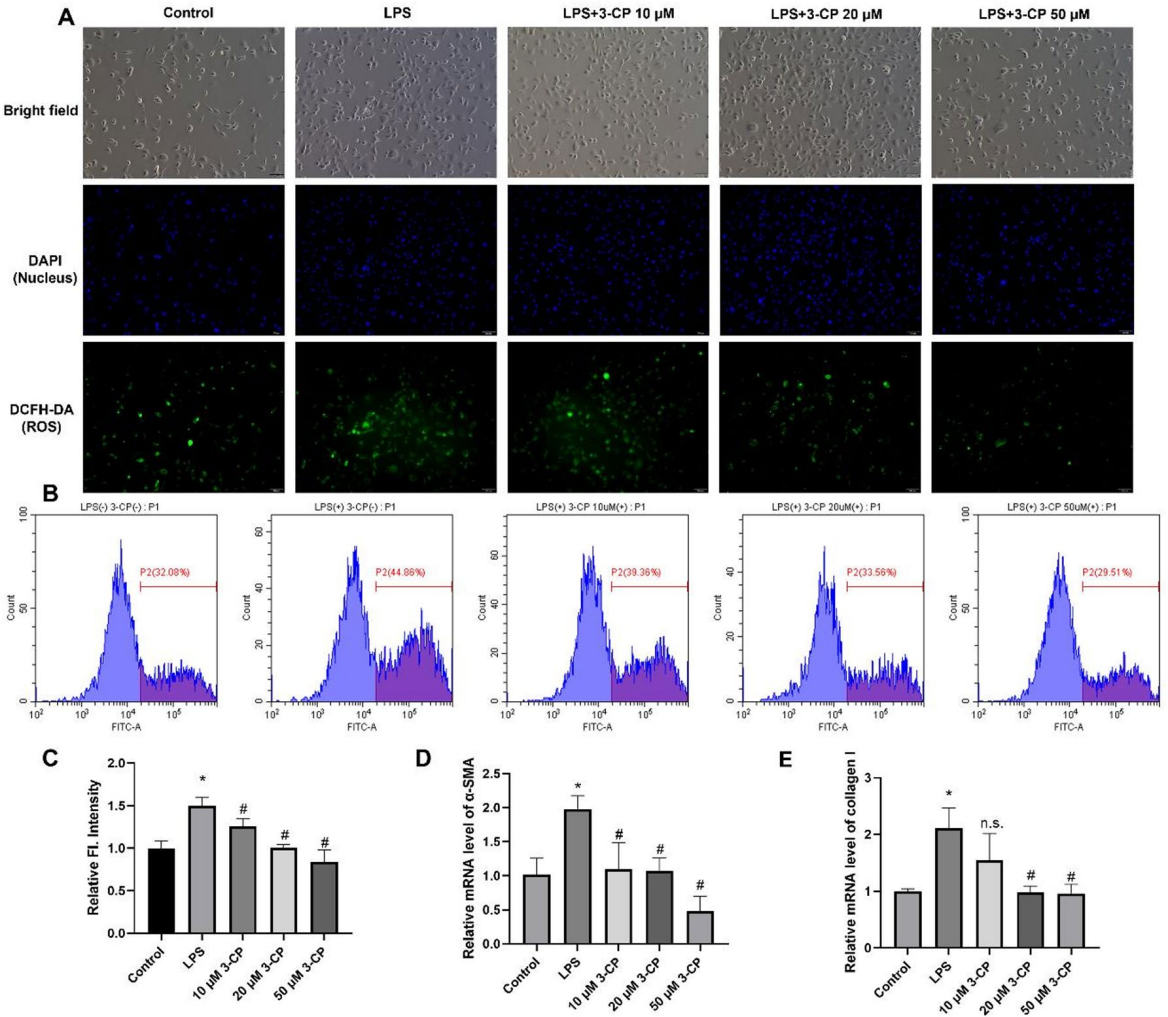
Effect of 3-CP on ROS content and activation of HSCs. (**A**-**B**) Fluorescence intensity of DCFH-DA in LX2 cells recorded by fluorescence microscope and flow cytometry (scar bar: 100 μm). (**C**) Relative fluorescence intensity of LX2 cells according to flow cytometry. Data are shown as mean ± SD, *n* = 3. *p < 0.05 vs. control group; #*p* < 0.05 vs. LPS group. (**D**-**E**) qRT-PCR analysis of the effects of 3-CP on the levels of α-SMA and type I collagen in LX2 induced by LPS. Data are shown as mean ± SD, *n* = 3. *p < 0.05 vs. control group; #*p* < 0.05 vs. LPS group.

ROS play a key role in the pathophysiology of liver fibrosis in both humans and animals. The effects of different concentrations of 3-CP (10, 20, and 50 µM) on ROS levels in hepatic stellate cells (LX2) stimulated with LPS (200 ng/mL) were studied using a DCFH-DA probe with both qualitative (fluorescence microscopy) and quantitative (flow cytometry) approaches. As shown in Fig. 2A-C, LPS stimulation significantly increased intracellular ROS levels compared to the control group. Importantly, 3-CP treatment dose-dependently attenuated this increase, with significant reductions observed even at 20 µM (*p* < 0.05 vs. LPS group). These findings indicated that nitroxide 3-CP effectively reduces ROS levels in activated HSCs.

### 3-CP inhibited the activation of HSCs.

To investigate the effect of 3-CP on LPS-induced liver fibrosis in LX2 cells, qRT-PCR was performed to measure α-SMA and COL1 expression levels. As Fig. 2D-E shows, LPS stimulation (200 ng/mL) significantly elevated α-SMA and COL1 mRNA levels in LX2 cells, suggesting that LPS potently activates HSCs. Conversely, 48-hour treatment with 3-CP (10, 20, and 50 µM) downregulated α-SMA and COL1 mRNA expression in a dose-dependent manner. These findings indicated that 3-CP suppresses HSC activation, consistent with its antioxidant effects shown in Fig. 2A-C.

### 3-CP has no effect on the proliferation of hepatocytes

To assess the safety of 3-CP in liver fibrosis treatment, specifically its effects on LX2 cell growth and normal hepatocyte growth, the CCK-8 assay was performed (Supplementary Fig. 1). When the concentration of 3-CP was lower than 200 µM, it had no effect on the L02 cells. Therefore, 3-CP is safe and can be used to treatment of liver fibrosis.

### 3-CP alleviated CCl_4_-induced liver fibrosis in mice

**Fig. 3:**
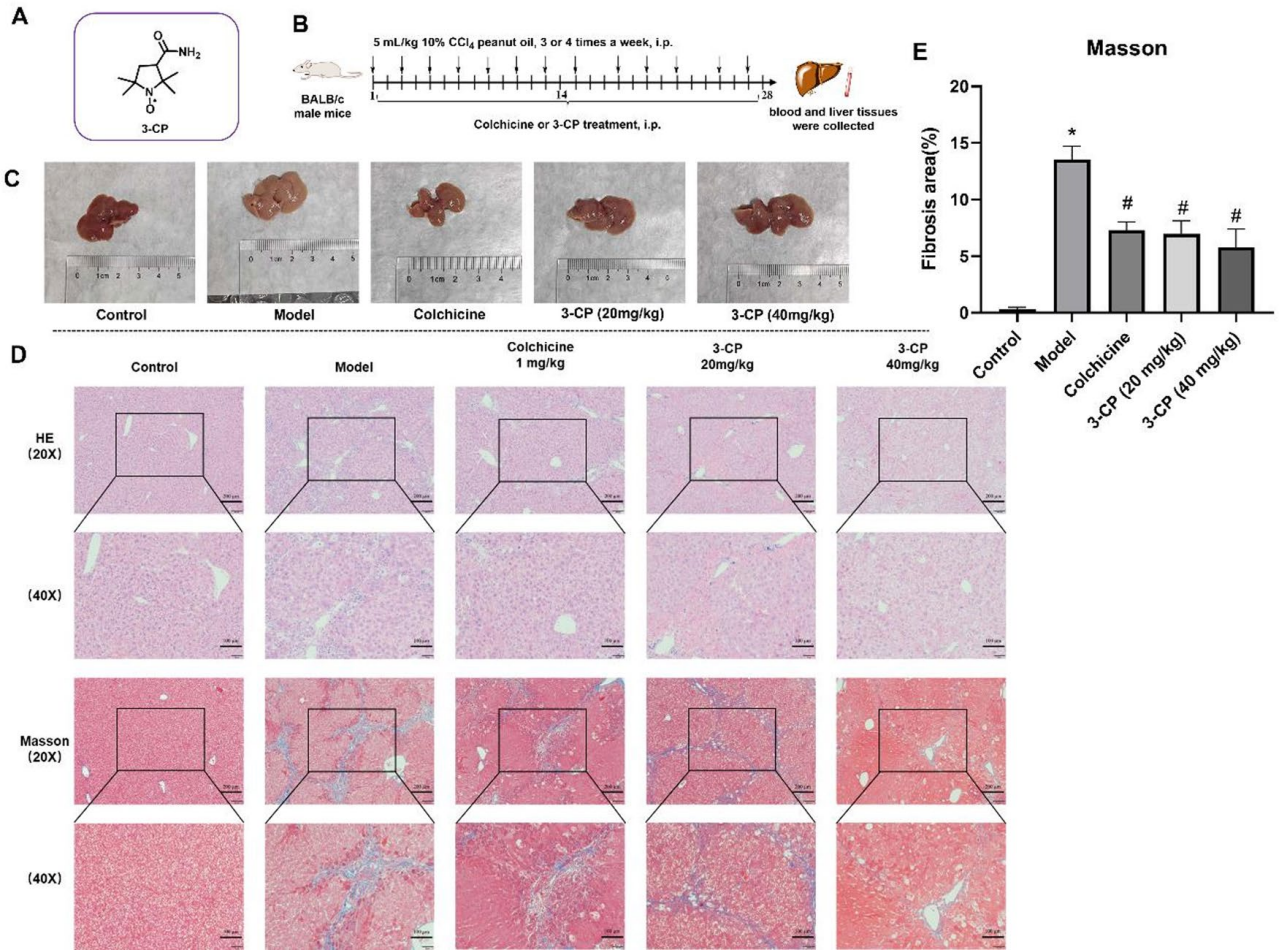
3-CP alleviated liver fibrosis in a CCl_4_-induced in mice. (**A**) Structure of nitroxide radical 3-CP. (**B**) Schematic diagram of the animal experiments. (**C**) Morphological observations of mouse liver. (**D**) Histological examination of liver slices by H&E and Masson’s trichrome staining (20x, scar bar: 200 μm; 40x, scar bar: 100 μm). (**E**) Fibrotic area (% field). Data are expressed as the mean ± S.D, *n* ≥ 6. *p < 0.05 vs. control group; #*p* < 0.05, vs. model group.

A mouse liver fibrosis model was established, as shown in Fig. 3. By observing the state and liver morphology of the mice, all the mice in the control group survived the experiment, showing sensitive responses, smooth fur, and good appetite. The morphology of the liver tissue from the dissected mice is shown in Fig. 3C. In the control group, the livers had a normal shape, were bright red in color, and had smooth surfaces. In the model group, the liver tissues were pale with rough and granular surfaces. Compared to the model group, liver color and morphology in the colchicine group, 3-CP low-dose group, and 3-CP high-dose group were markedly improved, with the 3-CP high-dose group showing more pronounced improvements. H&E and Masson staining of liver sections revealed that in the normal group, hepatocytes were neatly arranged with distinct hepatic lobule structures and normal cell morphology. In the model group, extensive hepatocyte necrosis; damage to the hepatic lobule, portal vein, and hepatic sinus structures; and prominent fibrous tissue proliferation were observed. Liver structural damage and fibrosis were significantly mitigated in the positive, low-, and high-dose groups with only minor collagen deposition. Fibrosis area was quantified from Masson-stained images using ImageJ software, with nine randomly selected fields analyzed per section in a blinded manner. After administering 3-CP of 20 and 40 mg/kg, the liver fibrosis area (% of the field) decreased from 13.6 ± 1.0% in the model group to 6.9 ± 0.9% and 5.7 ± 1.3%, respectively. These results suggest that 3-CP effectively alleviates CCl₄-induced liver fibrosis.

**Fig. 4:**
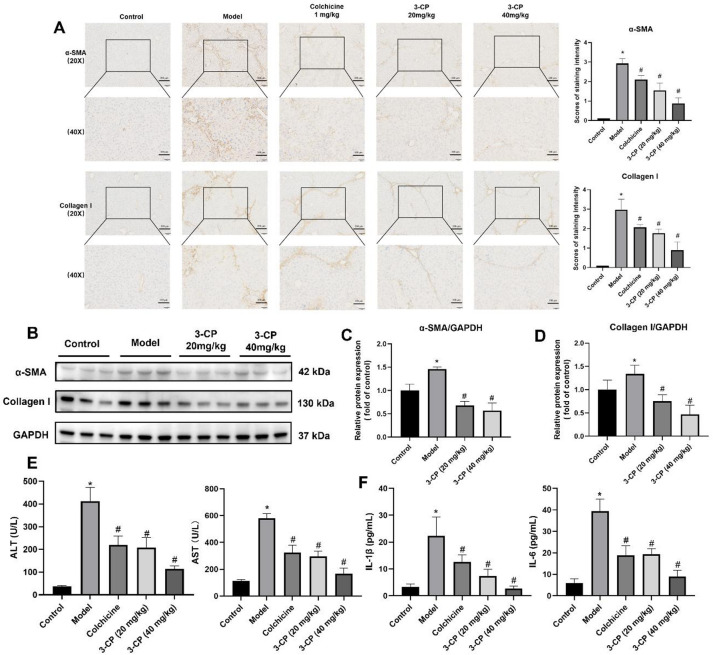
3-CP alleviated CCl_4_-induced liver fibrosis in mice. (**A**) Representative results and staining intensity scores of immunohistochemical staining forα-smooth muscle actin (α-SMA) and collagen I in the liver (20x, scar bar: 200 μm; 40x, scar bar: 100 μm). (**B**) Representative Western blot images showing protein expression of α-SMA, collagen I, and GAPDH. (**C**) Quantification of α-SMA protein expression. Data are presented as mean ± SD, *n* = 3. *p < 0.05, vs. control group; #*p* < 0.05, vs. model group. (**D**) Quantification of collagen I protein expression. Data are presented as mean ± SD, *n* = 3. *p < 0.05, vs. control group; #*p* < 0.05, vs. model group. (**E**) Serum index levels of liver function, and ALT and AST levels. (**F**) Levels of inflammatory factors (IL-1β and IL-6) were detected by ELISA. Data are presented as mean ± SD, *n* = 3. *p < 0.05, vs. control group; #*p* < 0.05, vs. model group.

Immunohistochemistry and western blotting results indicated that the expression levels of α-SMA and collagen I were elevated in the model group. In contrast, following 3-CP treatment (notably in 3-CP 40 mg/kg group), the protein levels of α-SMA and collagen I declined (Fig. 4A-D). Serum ALT and ALT/AST levels in mice in the CCl_4_ group were significantly increased (Fig. 4E). Notably, 3-CP treatment reversed this upward trend in the biochemical indices. As anticipated, ELISA results showed that compared with untreated mice, the levels of IL-1β and IL-6 in the CCl_4_ group were significantly increased, and 3-CP significantly reduced inflammatory infiltration, showing a decrease in the level of inflammatory factors (IL-1β/IL-6) (Fig. 4F). These results further demonstrated that 3-CP reduced the collagen content and alleviated CCl_4_-induced liver fibrosis in mice.

### 3-CP reduced liver inflammation in mice

**Fig. 5:**
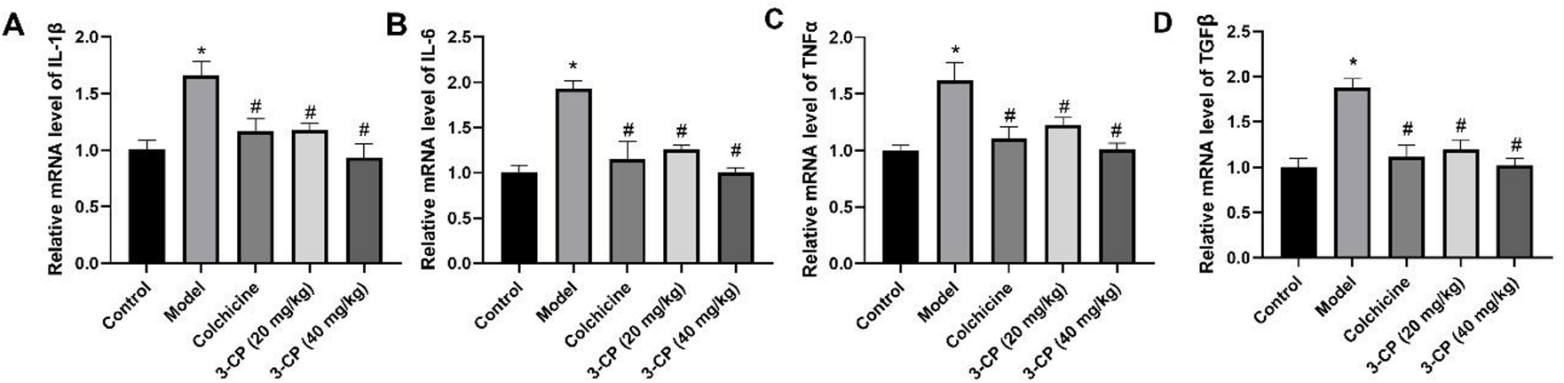
3-CP improves the inflammatory level of CCl_4_-induced liver fibrosis mice. (**A**-**D**) mRNA levels of inflammatory factors in the liver. Data are shown as mean ± SD, *n* = 3. *p < 0.05, vs. control group; #*p* < 0.05, vs. model group.

RT-PCR was used to measure the expression of inflammatory cytokines (IL-1β, IL-6, TNF-α, and TGF-β) in the liver tissue of each mouse group (Fig. 5). The results demonstrated that hepatic mRNA levels of all four cytokines were significantly elevated in the CCl_4_ model group compared to controls (*p* < 0.05). In contrast, treatment with 3-CP led to a notable reduction in their expression, ranging from approximately 35% to 55%. Following the administration of 3-CP, the expression of these inflammatory cytokines progressively reverted to the normal level. Collectively, these data demonstrate that 3-CP not only reduces collagen deposition but also suppresses the hepatic inflammatory response in CCl_4_-induced liver fibrosis, further supporting its therapeutic potential.

### 3-CP inhibited TLR4/NF-κB signaling pathway in liver fibrosis mice

Previous studies have established the critical role of the TLR4/NF-κB signaling pathway in the pathogenesis of liver fibrosis. LPS activates this pathway primarily through the MyD88-dependent route, leading to NF-κB activation, which in turn promotes HSC activation and perpetuates chronic liver injury^37^.

**Fig. 6:**
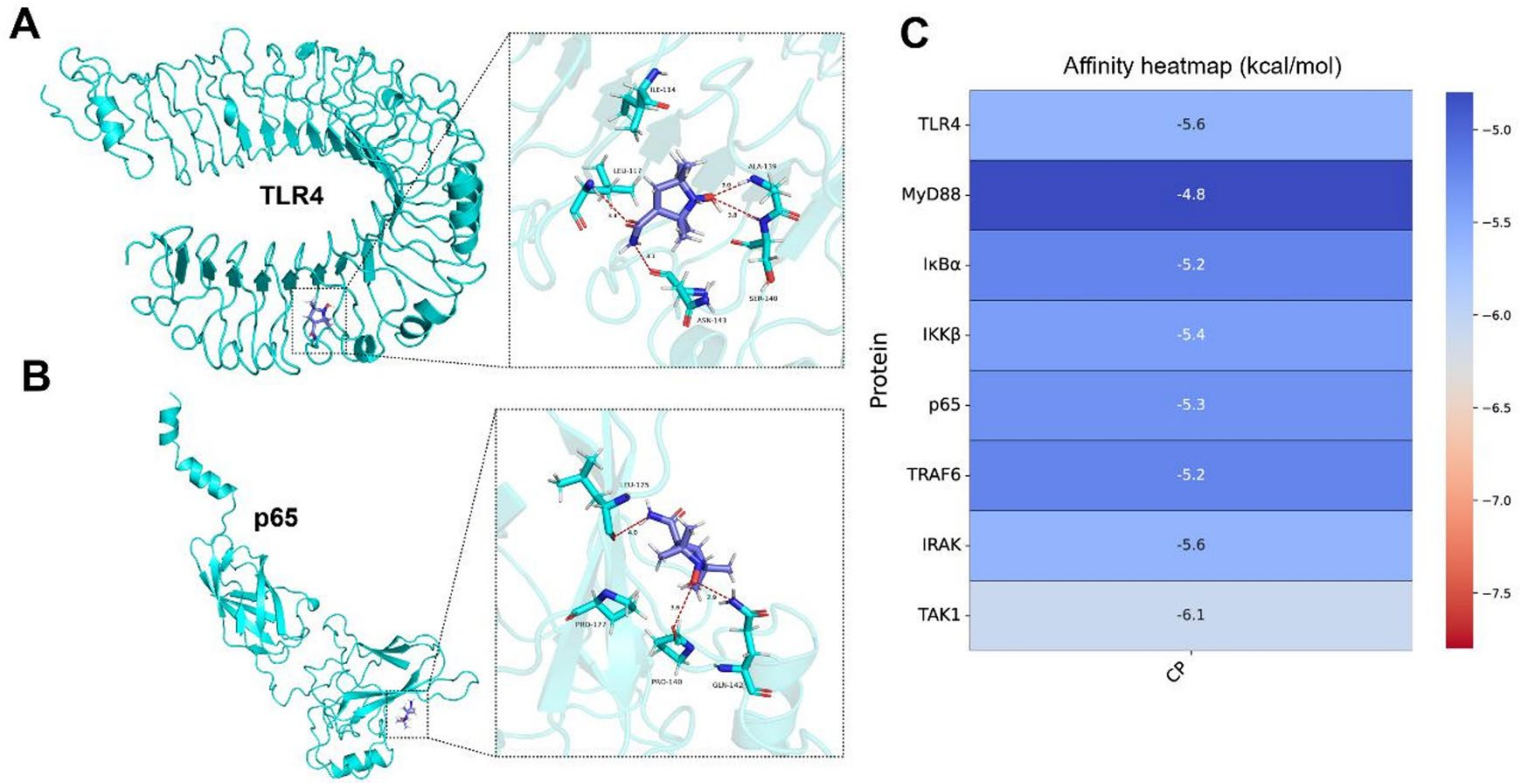
Molecular docking analysis of 3-CP with TLR4 and p65. (**A**-**B**) Predicted 3D binding model of 3-CP and TLR4/p65. 3-CP is colored purple. The protein surfaces of TLR4/p65 are colored in cyan. Potential interactions are indicated by dashed lines. (**C**) Predicted protein-ligand binding affinities (kcal/mol).

To explore whether 3-CP exerts its anti-fibrotic effects through modulation of this pathway, we performed molecular docking analysis to predict potential interactions between 3-CP and key proteins within the TLR4/MyD88/NF-κB signaling cascade. The molecular docking results for 3-CP with TLR4 and p65 are shown in Fig. 6, and predicted binding models with MyD88,IκBα and IKKβ are presented in Supplementary Fig. 2. According to vina’s prediction algorithm of binding energy based on AMBER force field, the binding energy between 3-CP and TLR4/p65 is predicted to be -5.6 kcal/mol and − 5.3 kcal/mol, respectively (Fig. 6C). A certain number of hydrogen bond interactions were formed between the protein and 3-CP; the specific interacting residues are shown in Fig. 6A-B. The binding energies and key residues for all targets are listed in Supplementary Table S2. Therefore, these in silico predictions suggest that TLR4, MyD88, IκBα, IKKβ, and p65 may be potential interacting partners of 3-CP within this signaling pathway. However, it is important to note that these docking results are exploratory and hypothesis-generating; they provide preliminary indications but do not constitute definitive evidence of direct binding. Further biochemical validation is required to confirm these interactions.

**Fig. 7:**
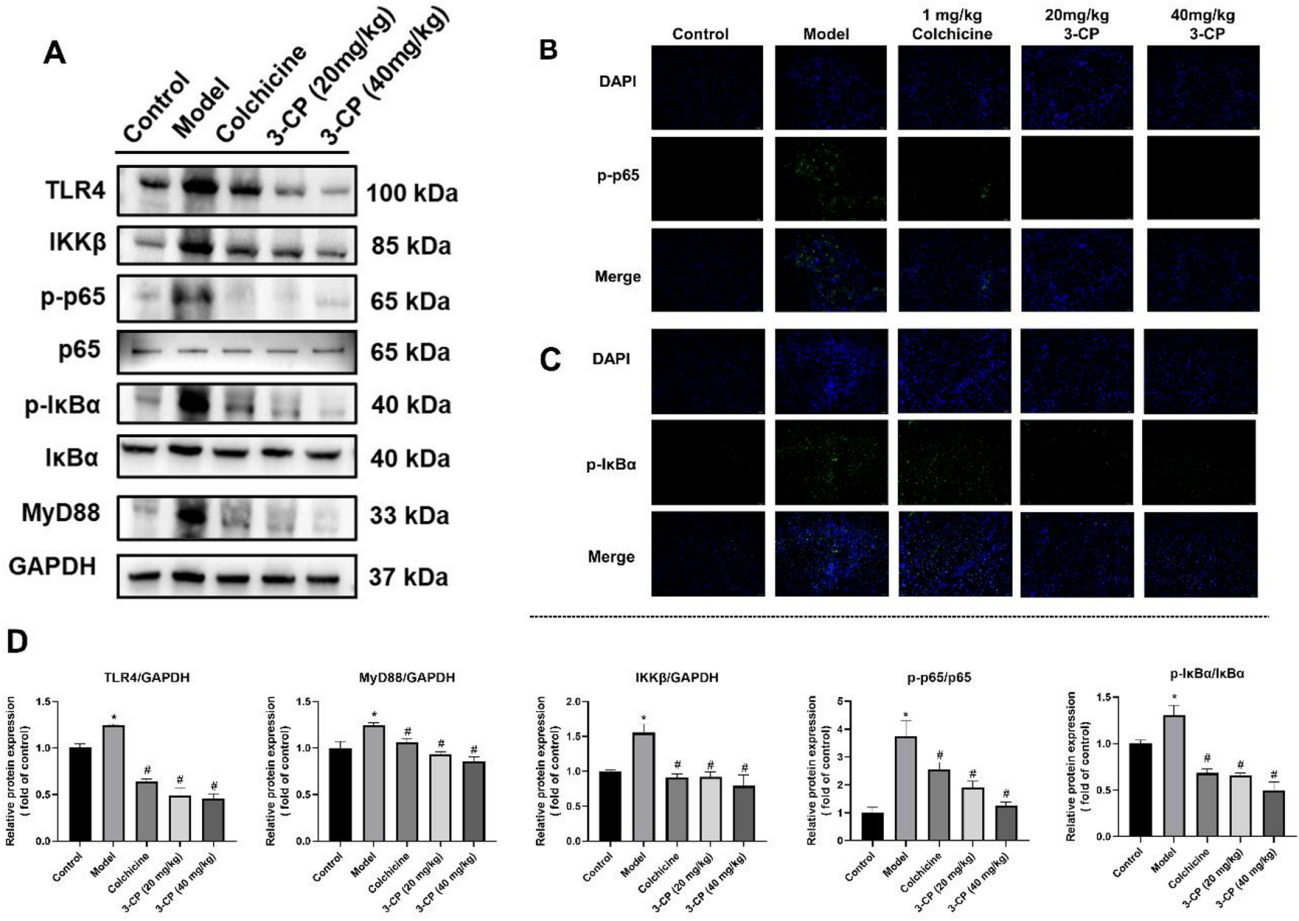
3-CP is associated with inhibition of the TLR4/NF-κB pathway in the CCl_4_-induced liver fibrosis mice model. (**A**-**B**) Protein expression levels of TLR4/NF-κB signaling-associated proteins (TLR4, MyD88, IKKβ, p65/p-p65 and IκBα/p-IκBα). (**B**-**C**) Representative results of immunofluorescence staining for p-p65 and p-IκBα (scale bar: 50 μm). (**D**) Quantification of TLR4, MyD88, IKKβ, p65/p-p65 and IκBα/p-IκBα protein expression. Data are presented as mean ± SD, *n* = 3. *p < 0.05, vs. control group; #*p* < 0.05, vs. model group.

To investigate the molecular mechanism underlying the anti-fibrotic effects of 3-CP, we examined proteins related to the TLR4/NF-κB signaling pathway, including TLR4, MyD88, IKKβ, p65, and IκBα. Western blot analysis showed that protein levels of TLR4, MyD88, IKKβ, phosphorylated p65 (p-p65), and phosphorylated IκBα (p-IκBα) were significantly elevated in the CCl_4_-induced liver fibrosis model. However, after 3-CP treatment, the levels of these activated pathway components decreased (Fig. 7), as shown in Fig. 7A. Specifically, 3-CP administration reduced TLR4, MyD88, IKKβ, p-p65, and p-IκBα levels relative to the model group.

These findings were further confirmed by immunofluorescence analysis (Fig. 7B-C), which revealed increased fluorescence intensity for p-p65 and p-IκBα in liver tissues from CCl_4_-treated mice, whereas 3-CP treatment markedly attenuated these signals. Collectively, these results demonstrate that 3-CP treatment is associated with inhibition of the TLR4/NF-κB signaling pathway in the CCl_4_-induced liver fibrosis model.

In summary, the present study demonstrates that 3-CP exhibits potent anti-inflammatory and anti-fibrotic activity in both in vitro and in vivo models. Our findings indicate that 3-CP suppresses LPS-induced pro-inflammatory responses and LX2 cell activation, and effectively attenuates CCl_4_-induced liver fibrosis in mice. These effects are associated with inhibition of the TLR4/NF-κB signaling pathway, suggesting that modulation of this pathway may contribute to the therapeutic action of 3-CP.

## Discussion

Most chronic liver diseases of various etiologies manifest as liver fibrosis, a pathological process involving multiple intrahepatic cell types^38^. Hepatic stellate cells (HSCs) are of particular significance, as they serve as the ultimate effector cells targeted by various fibrogenic factors^39^. In the CCl_4_-induced mouse model of liver fibrosis, ample evidence suggests that oxidative stress is a key element in the pathophysiological process. The generation of reactive oxygen species (ROS) not only directly damages hepatocytes but also induces oxidative stress, disrupting the connections between endothelial cells^40^. This facilitates migration of inflammatory cells across the endothelial barrier, ultimately leading to liver injury. After liver injury, quiescent hepatic stellate cells (HSCs) are activated into myofibroblasts, producing abundant ECM components such as α-SMA and COL1^14,41^. This disrupts the ECM synthesis-degradation balance, causing ECM deposition and subsequent liver damage. In both the LPS-induced HSC model and the CCl_4_-induced liver fibrosis murine model, we observed elevated ROS levels and increased inflammation. Histological examination of the liver interstitium revealed disrupted lobular architecture, extensive hepatocyte necrosis, and notable collagen deposition, confirming successful establishment of the liver fibrosis model in this study.

For an extended period, nitroxide radicals have been recognized for their ability to protect experimental animals from oxidative damage and inflammation^30,42^. It has been hypothesized that nitroxide radicals act as catalytic antioxidants that reduce tissue damage under inflammatory conditions. This is primarily due to their rapid reaction with free radicals, which significantly reduces the ROS levels of reactive oxygen species. Pirfenidone, an anti-fibrotic drug currently used primarily for idiopathic pulmonary fibrosis, has been suggested to mitigate oxidative stress damage in hepatocytes through free radical scavenging.^43^ Similar to pirfenidone, 3-CP possesses a low molecular weight and favorable oil-water partition coefficient, enabling it to penetrate cell membranes and reach intracellular oxidative stress sites, potentially offering broader antioxidant protection. In addition, we observed that 3-CP decreased the mRNA levels of α-SMA and type I collagen in LPS-stimulated LX2 cells, suggesting that 3-CP inhibits HSC activation. These in vitro findings were further corroborated by our in vivo experiments demonstrating the inhibitory effects of 3-CP on HSC activation and liver fibrosis.

In our study, 3-CP treatment markedly improved liver tissue morphology in CCl_4_-induced liver fibrosis, decreased serum levels of the liver function markers ALT and AST, and reduced expression of α-SMA and type I collagen. Hematoxylin-eosin and Masson’s trichrome staining revealed that 3-CP alleviated liver fibrosis and abnormal collagen deposition in mice. PCR results showed that 3-CP significantly reduced the hepatic mRNA levels of IL-1β, IL-6, TNF-α, and TGF-β compared with the model group. Obeticholic acid, an farnesoid X receptor (FXR) agonist, primarily targets the hepatobiliary system to regulate bile acid metabolism and alleviate liver inflammation^44^. In contrast, 3-CP appears to have a broader anti-inflammatory spectrum, potentially acting on various inflammatory cells and tissues, which may benefit not only liver but also other inflammatory conditions (Supplementary Table S3). While pirfenidone and obeticholic acid have established therapeutic roles, our in vitro and in vivo findings suggest that 3-CP’s combined antioxidant and anti-inflammatory effects may offer a unique and potentially more comprehensive strategy for treating diseases involving oxidative stress and inflammation.

Previous reports have indicated that inhibition of the NF-κB signaling pathway is crucial for alleviating liver fibrosis^6^. In hepatocytes, appropriate activation of NF-κB enhances cell survival and anti-apoptotic capabilities^45^. However, excessive or insufficient activation can exacerbate inflammatory response^46^. Excessive activation may lead to further cell damage, whereas insufficient activation may not offer effective protection against cell death. In hepatic stellate cells (HSC), cell activation triggered by injury is mediated by NF-κB activation of NF-κB^47^. NF-κB is a crucial regulator of HSC survival and development of fibrosis in chronic liver diseases. The NF-κB protein family consists of RelA (p65), RELB, C-REL, p50 ( P105 precursor), and p52 ( p100 precursor)^48^. The TLR4/MyD88 signaling pathway mainly mediates inflammatory responses. LPS activates the TLR4/NF-κB pathway via two main routes, with the MyD88-dependent pathway, the most classical one in this experiment, being one of them^37^. In this study, p65 was selected as representative of NF-κB. NF-κB regulates IκBα transcription, while IκBβ controls NF-κB inactivation. Upon generation of inflammatory signals, cytoplasmic IκBα is phosphorylated and degraded, allowing the NF-κB protein to enter the nucleus^49^. The IKK complex, a key molecule in activating the NF-κB signaling pathway, targets IκBα to release NF-κB and initiate the NF-κB signaling cascade^50^.

In this study, exploratory molecular docking was used to investigate potential interactions between 3-CP and components of the TLR4/NF-κB signaling pathway. It is important to emphasize that these in silico predictions are hypothesis-generating and do not constitute evidence of direct binding. The docking results were further supported by western blotting and immunofluorescence experiments. The results showed that 3-CP suppressed IKKβ expression, resulting in reduced p65 release. It also inhibited the expression of phosphorylated p65 (p-p65), a marker of NF-κB activation. In the model group, IκBα protein expression decreased compared to controls, indicating increased p65 levels, consistent with the p65 assay results. Notably, 3-CP significantly inhibited p-IκBα expression, suggesting reduced p65 release and consequent alleviation of inflammation. These findings are consistent with the PCR results. Collectively, these data suggest that the anti-inflammatory effects of 3-CP may be associated with regulation of the NF-κB signaling pathway through inhibition of IKKβ, p65, p-p65, and p-IκBα, which aligns with the immunofluorescence results.

While our findings demonstrate an association between 3-CP treatment and inhibition of the TLR4/NF-κB pathway, several limitations must be acknowledged. First, the lack of pathway-blocking experiments (e.g., using TLR4/NF-κB inhibitors or genetic approaches) means the current data remain correlative and cannot establish definitive causality. Gene-knockout animals or those fed with specific inhibitors of 3-CP’s suspected targets should be used to confirm if 3-CP’s effects (e.g., anti-inflammatory, anti-oxidation) are related to regulating these targets. Second, the molecular docking results are exploratory and require biochemical validation (e.g., SPR, CETSA) to confirm direct target engagement, as the moderate binding energies and suggestion of multiple targets raise specificity concerns. Third, the brief in vivo observation period (4 weeks) precludes assessment of long-term safety, pharmacokinetics, or tissue distribution. Fourth, in vitro experiments lacked positive controls, assessment of ROS levels in hepatocytes, and mechanistic studies to explain plateau effects observed in apoptosis and gene expression assays. Finally, the absence of comparative studies in other fibrotic models and pharmacokinetic profiling limits broader therapeutic interpretation. Therefore, future studies employing pathway-specific inhibitors, genetic approaches, biochemical binding assays, and long-term toxicity assessments are essential to validate whether TLR4/NF-κB signaling is causally required for the anti-fibrotic effects of 3-CP and to establish its true therapeutic potential.

## Conclusion

Liver fibrosis is a prevalent pathological feature in most chronic liver diseases. The activation and transformation of HSCs into myofibroblasts are the main drivers of liver fibrosis. This study confirmed that 3-CP significantly inhibits HSC activation, as demonstrated by both in vivo and in vitro experiments. Specifically, 3-CP reduced the mRNA levels of α-SMA and collagen I, improved liver tissue morphology in CCl₄-induced liver fibrosis, and decreased the levels of liver function markers. Mechanistically, the anti-inflammatory effects of 3-CP are associated with modulation of the NF-κB signaling pathway, as evidenced by suppressed expression of MyD88, IKKβ, p65, p-p65, and p-IκBα. Collectively, these results position 3-CP as a promising therapeutic candidate for liver fibrosis, with its dual antioxidant and anti-inflammatory actions offering a unique approach compared to existing agents. Further studies are needed to evaluate the long-term safety, pharmacokinetic profile, and therapeutic efficacy of 3-CP in preclinical models before any potential clinical application can be considered.

## Materials and methods

### Reagents

3-CP (purity > 99%; C5151), colchicine (C3915), lipopolysaccharide LPS (Escherichia coli 055, B5; L2880), dimethyl sulfoxide (DMSO; D8418), and 2’,7’-dichlorofluorescein diacetate (DCFH-DA; D6883) were purchased from Sigma-Aldrich (Shanghai, China). Immortalized human hepatic stellate cells LX2 (FH0108) and human hepatocytes L02 (FH0109) were purchased from the Enrich Cell Center (Shanghai, China). Fetal bovine serum (FBS; A5670701), high-glucose Dulbecco’s Modified Eagle’s medium (DMEM), and 0.25% trypsin-ethylenediaminetetraacetic acid (trypsin-EDTA) were obtained from Gibco Life Technologies (Carlsbad, CA, USA). Primary antibodies against NF-κB p65 (Cat #8242S), α-SMA (Cat# 19245 S), collagen I (Cat# 72026 S) and secondary antibody anti-rabbit IgG (CST#7074S) were purchased from Cell Signaling Technology (Danvers, MA, United States). Primary antibodies against TLR4 (Cat#AF7017), myeloid differentiation factor 88 (MyD88, Cat#AF5195), p-p65 (Cat#AF2006), IκBα (Cat# AF5002), p-IκBα (Cat#AF2002), and IKKα/β (Cat#AF6014) were provided by Affinity Biosciences Co., Ltd. (Jiangsu Province, China). The primary antibody targeting GAPDH was supplied by Wuhan Servicebio Technology Co. Ltd. (Hubei Province, China). Cell Counting Kit-8 (CCK-8), Annexin V-APC/7-AAD apoptosis detection kit, RIPA lysis buffer, and bicinchoninic acid assay (BCA) protein assay kits were obtained from Fushen Biotechnology Co., Ltd. (Shanghai, China). The One-Step TB Green Prime Script PLUS Real-Time Quantitative PCR (RT-PCR) Kit (RR096A) was obtained from Takara Bio Inc. (Otsu, Shiga, Japan). Mouse IL-1α and mouse IL6 Elisa kits were acquired from Multi Sciences Biotech Co. Ltd. (Hangzhou, China).

### Cell culture and proliferation assay

Cells were cultivated in high-glucose DMEM supplemented with 1% penicillin-streptomycin and 10% fetal bovine serum (FBS). The culture dishes were placed in a thermostatically controlled incubator maintained at 37 °C in a 5% CO₂ atmosphere. LX2 cells were used within passages 5–15, and L02 cells were used within passages 4–12 to ensure consistent phenotypic characteristics. Once the cells achieved 80–90% confluence or were deemed suitable for digestion, they were subcultured. Subsequently, the subcultured cells were preserved in a freezer set at -80 °C for subsequent experimental procedures.

LX2 and L02 cell proliferation was evaluated using the CCK-8 assay following the manufacturer’s instructions. Cells were seeded in 96-well plates at a density of 5 × 10³ cells/well. Cells were stimulated with 200 ng/mL LPS (for LX2) and treated with 3-CP (0, 5, 10, 20, 30, 40, 50, 100, and 200 µM) for 48 h. After treatment, 10 µL CCK-8 was added to each well. Four hours later, absorbance was measured at 450 nm using a microplate analyzer (Envision, PerkinElmer, USA). Cell viability (%) was calculated as follows: (OD of experimental group/OD of control group)×100.

### Transwell migration assay

LX2 cells were seeded in 24-well plates at 8 × 10⁴ cells/well and incubated for 24 h. The cells were then treated with 200 ng/mL LPS and 3-CP (0, 10, 20, and 50 µM) for 2 days. Transwell chambers (Corning, Corning, NY, USA) were placed in 24-well plates. A single-cell suspension (3 × 10⁴ cells) in serum-free medium was added to the upper chamber and 600 µL of medium with 10% fetal bovine serum was added to the lower chamber. LX2 cells on the bottom side of the transwell chamber were fixed with paraformaldehyde and stained with crystal violet for 24 h. Images were captured from five randomly selected fields per well at 200× magnification using a fluorescence microscope (Olympus, Japan). Migrated cells were counted blindly by two independent researchers using ImageJ software (cell counter plugin, version 1.53) to ensure unbiased quantification. Before counting, uniform cell distribution across the membrane was verified by visual inspection, and only wells with even staining were included in the analysis.

### Cell cycle and apoptosis

LX2 cells were cultured and treated as described previously. For apoptosis detection, the culture medium was removed, and 3–5 × 10⁵ cells were collected and washed. Next, Annexin V-allophycocyanin (Annexin V-APC) and 7-amino-actinomycin D (7-AAD) staining solutions were added to the cell suspension. The mixture was incubated in the dark, followed by adding 400 µL of 1× binding buffer. Samples were analyzed by flow cytometry (Beckman CytoFLEX LX, China) within 1 h, and data were processed using FlowJo v7.6 (FlowJo LLC, Ashland, OR, USA). Apoptotic cells were quantified as the sum of early (Annexin V+/7-AAD-) and late (Annexin V+/7-AAD+) apoptotic populations.

### ROS assay

LX2 cells were cultured and treated as described previously. DCFH-DA was diluted 1:1000 in serum-free medium to a final concentration of 10 µM. The LX2 cells were incubated with DCFH-DA for 30 min at 37 °C in the dark. Subsequently, the cells were washed three times with serum-free cell culture medium to completely remove the DCFH-DA. Intracellular ROS levels were assessed by both qualitative fluorescence microscopy and quantitative flow cytometry. For microscopy, images were captured from five random fields per well (Olympus, Japan). For flow cytometry, analyses were performed within 3 h (Beckman CytoFLEX LX, China), with 10,000 events recorded per sample. Mean fluorescence intensity was calculated to quantify ROS levels.

### Animals and in-vivo experiments

Male BALB/c mice (6–8 weeks old; 18–22 g) were obtained from the Jiesijie Laboratory Animal Co., Ltd. They were housed in an animal room at a standard temperature of 21 ± 2 °C and relative humidity of 50 ± 10%. After 7 days of adaptive feeding, experiments were conducted. All animal experimental protocols were performed in accordance with relevant guideline and regulations and were approved by the Animal Ethics Committee of Ninth People’s Hospital, Shanghai Jiao Tong University School of Medicine (Approval No.: SH9H-2023-A911-1). The animal experiments in this study were performed in accordance with the ARRIVE guidelines.

This experiment was performed according to a previously reported model^51^. Mice were randomly divided into 5 groups (*n* = 6): (1) the control group, mice were treated with 2 mL/kg 5% peanut oil three times per week for 4 weeks; (2) the fibrotic group (model), mice were intraperitoneally injected with 2 mL/kg 5% CCl₄-peanut oil three times per week for 4 weeks; (3) the positive drug group (colchicine), mice were treated with 2 mL/kg 5% CCl₄-peanut oil three times per week for 4 weeks and 0.1 mL 1 mg/kg colchicine solution^52^; (4) the low 3-CP treated group (3-CP 20 mg/kg), mice were treated with 2 mL/kg 5% CCl₄-peanut oil three times per week for 4 weeks and 0.1 mL 20 mg/kg 3-CP solution; (5) the high 3-CP treated group (3-CP 40 mg/kg), mice were treated with 2 mL/kg 5% CCl₄-peanut oil three times per week for 4 weeks and 0.1 mL 40 mg/kg 3-CP solution. The doses of 3-CP (20 and 40 mg/kg) were selected based on previous studies as they demonstrated significant and dose-responsive reductions in fibrosis markers without observable toxicity^53^. The 3-CP and colchicine were dissolved in distilled water. The fibrotic group received intraperitoneal CCl₄ for 4 weeks, while treatment groups received 3-CP or colchicine at different doses via intraperitoneal injection for 4 weeks, ensuring model establishment, preventing mouse death, and reducing pain. Mice had free access to a normal diet and were maintained under ambient temperature and light conditions. After four weeks of treatment, all BALB/c mice were anesthetized with 2% isoflurane at a 0.5-mL/min gas flow rate and then euthanized by cervical dislocation. Blood was collected and the liver tissues were dissected. Part of the liver tissue was fixed in 4% paraformaldehyde for chromoscopy, while the remaining tissue was stored at -80 °C.

### Biochemical evaluation

Blood was centrifuged at 1000 g for 15 min to obtain serum. Following the manufacturer’s protocol, ALT and AST activities were assayed using an automatic biochemical analyzer (Chemray 240, Kayto, Shenzhen, China).

### ELISA detection of pro-inflammatory cytokines

Following the manufacturer’s protocol, ELISA kits were used to measure serum levels of pro-inflammatory IL-1α and IL-6. A microplate reader (Envision, PerkinElmer, USA) was used to detect absorbance at 450 and 570 nm.

### Immunocytochemical technique and histological analysis

Fresh liver tissues were fixed in neutral formalin and embedded in paraffin. Five-micrometer paraffin sections were prepared and stained with hematoxylin (eosin) and Masson’s trichrome^54^. Histological and pathological changes were observed using a light microscope (Nikon E100, Japan). The area of hepatic fibrosis was quantified from Masson-stained images using ImageJ software (version 1.53), with nine randomly selected fields per section analyzed in a blinded manner by two independent researchers.

For immunohistochemistry, paraffin-embedded liver and intestinal 4-µm sections were incubated overnight with anti-α-SMA and anti-collagen I antibodies. Subsequently, the sections were incubated with horseradish peroxidase (HRP)-conjugated secondary antibody for 60 min, stained with diaminobenzidine (DAB) for 15 min, and restained with hematoxylin. Sections were observed under a light microscope (Nikon E100, Japan). For immunofluorescence staining, antigens in the sections were repaired, blocked with bovine serum albumin, and treated with anti-p-p65 and anti-p-IκBα antibodies. The sections were then incubated with fluorescently labeled secondary antibodies and counterstained with DAPI for nuclear staining. All liver tissue sections were scanned using a Panoramic MIDI scanner (3D Histech, Hungary) and evaluated using the Case Viewer 2.4 (3D Histech, ).

### RT-PCR

Total RNA was extracted from cells and tissue samples using TRIzol reagent (Invitrogen, USA) following the manufacturer’s protocol. The purity and concentration of the extracted RNA were assessed using Nanodrop 2000 (Thermo Fisher Scientific, USA), and then reverse-transcribed into cDNA. RT-PCR was used to detect mRNA expression levels of human α-SMA and collagen I (for in vitro studies), and mouse interleukin-1β (IL-1β), interleukin-6 (IL-6), tumor necrosis factor-α (TNF-α), and tumor growth factor-β (TGF-β) using a Light Cycler 480II system (Roche Pharmaceuticals, Switzerland). Amplification and melting curves were generated, with GAPDH serving as the internal control. Data were calculated from the amplification curve using the 2^–ΔΔCt^ formula^55^. Primer sequences used are listed in Supplementary Table 1.

### Western blot analysis

Total protein was extracted from liver tissues. A standard curve was plotted using a BCA protein assay kit following the manufacturer’s protocol to determine the protein content of the samples. Standardized protein samples were separated using polyacrylamide gel electrophoresis and transferred to a PVDF membrane at 220 mA for 100 min. After the membrane was treated with 5% skim milk sealing solution, it was incubated overnight with rabbit anti-TLR4, MyD88, p65/p-p65, IKKα/β, IκBα/p-IκBα, α-SMA, type I collagen and GAPDH (dilution ratio 1:1000), and then the membrane was rinsed and coupled with goat anti-rabbit secondary antibody (dilution ratio 1:5000). Glyceraldehyde-3-phosphate dehydrogenase was used as the internal reference. Band intensities were quantified using ImageJ software (version 1.53) with GAPDH as the loading control. All western blot experiments were performed in triplicate.

### Molecular docking

Molecular docking was used to explore potential interactions between 3-CP and key proteins in the TLR4/NF-κB signaling pathway. Based on previous studies, proteins (TLR4, MyD88, IκBα, IKKβ, p65, TRAF6, IRAK, and TAK1) in the signal transduction network were chosen as research targets^56^. The 3D structures of these target proteins were downloaded from the Protein Data Bank (PDB). 3-CP was imported into Discovery Studio 3.5 software for hydrogenation optimization and docked with the target protein. Blind docking was performed via an CB-DOCK2 online server. CB-DOCK2 detects cavities using an artificial neural network and performs docking using Autodock Vina^57^. To validate the docking reliability, the co-crystallized ligand was re-docked into the binding site of each target protein where available, and the root mean square deviation (RMSD) between the predicted and experimental binding poses was calculated. An RMSD value < 2.0 Å was considered acceptable, confirming that the docking parameters were appropriate for reproducing native binding modes. The PLIP online server (https://plip-tool.biotec.tu-dresden.de/plip-web) was used to analyze the interaction forces between the ligand and receptor, and PyMol 2.2 (Schrödinger LCC, New York, NY, USA) was used to visualize the 3D conformation of the ligand-receptor complex. These docking results are exploratory and hypothesis-generating, providing preliminary predictions that require experimental validation to confirm direct binding interactions.

### Statistical analysis

Statistical analyses were conducted using SPSS software version 26 (SPSS Inc., Chicago, IL, USA). Data are presented as the mean ± standard deviation. One-way ANOVA was employed to assess differences among multiple comparisons, followed by the Student-Newman-Keuls post hoc test. Statistical significance was defined as *p* < 0.05. All data were analyzed using GraphPad Prism 9.0 (GraphPad Software Inc., USA).

## Supplementary Information

Below is the link to the electronic supplementary material.

Supplementary Material 1

## Data Availability

The generated and/or analysed during the current study are available in the Fig.share repository, [doi: 10.6084/m9.Fig.share.29606744].
