# Supplementary material for: 3-carbamoyl proxyl nitroxide attenuates CCl4-induced liver fibrosis in mice through antioxidant-inflammatory regulation of TLR4/NF-κB signaling pathway
Source: Sci Rep. 2026 Mar 27;16:10798. doi: 10.1038/s41598-026-46137-1 (PMC13039547; doi:10.1038/s41598-026-46137-1)
Supplement: Supplementary file 1 — Supplementary Material 1 [file 41598_2026_46137_MOESM1_ESM.docx]

**Supplementary Material**

1. **Supplementary Figures**

**Supplementary Fig.1.** Effects of different concentrations of 3-CP on L02 cells. Experimental data are presented as mean ± SD, n=6 (n.s. represents no significance).


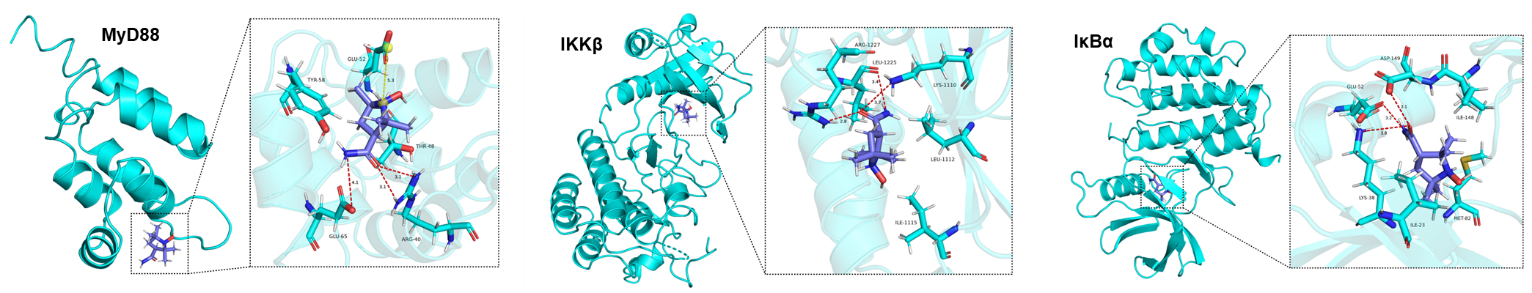


**Supplementary Fig. 2.** The 3D binding model of 3-CP and MyD88/IκBα/IKKβ. The 3-CP is colored in purple. The surface of MyD88/IκBα/IKKβ is colored in cyan. The interactions are depicted as dashed lines.

**Original blot for Figure 4B：**

α-SMA:


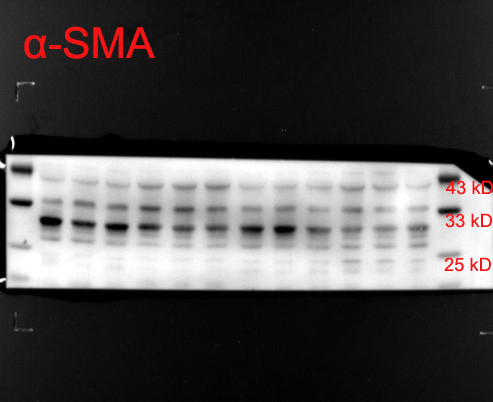


Collagen I:


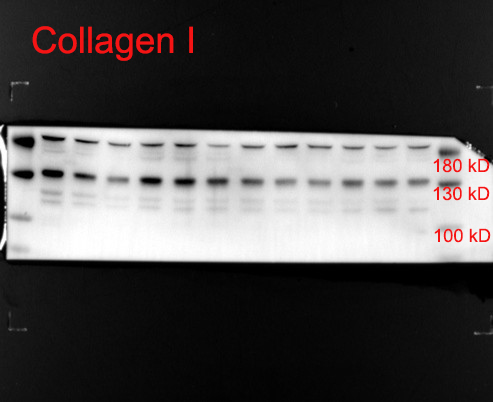


GAPDH:


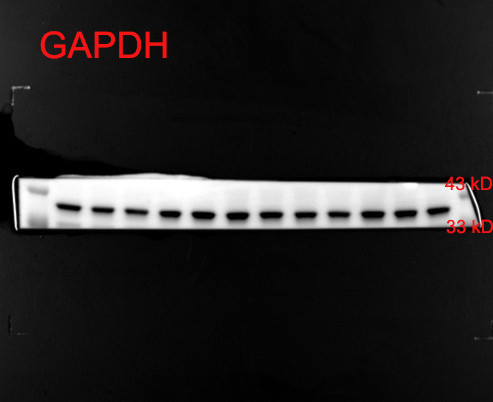


**Original blot for Figure 7A：**

TLR4:


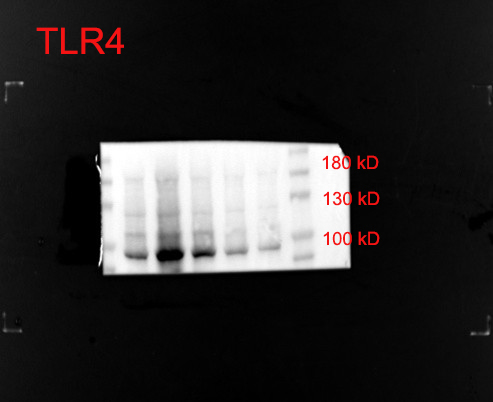

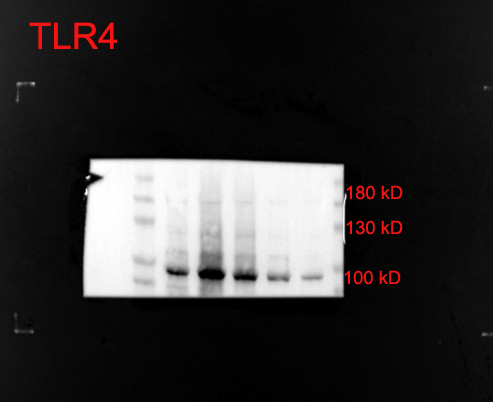

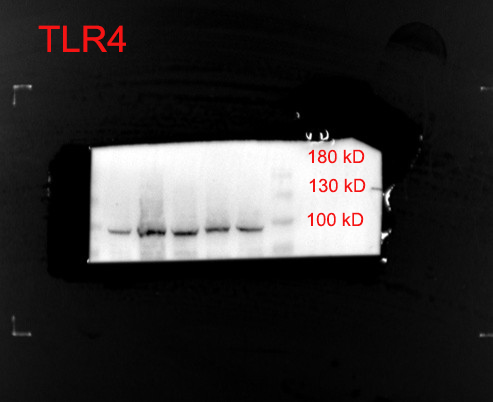


IKKβ:


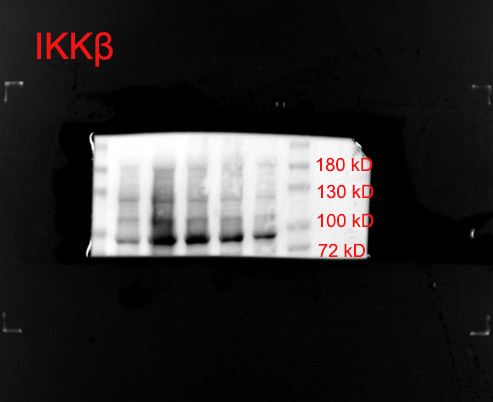

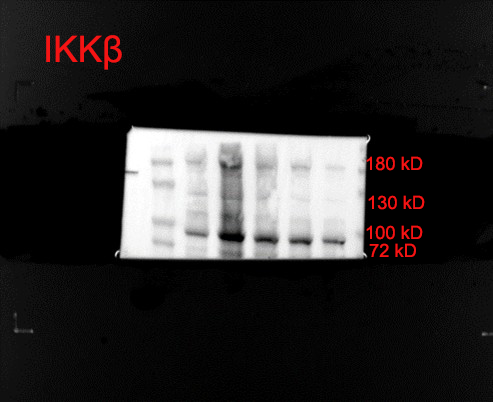

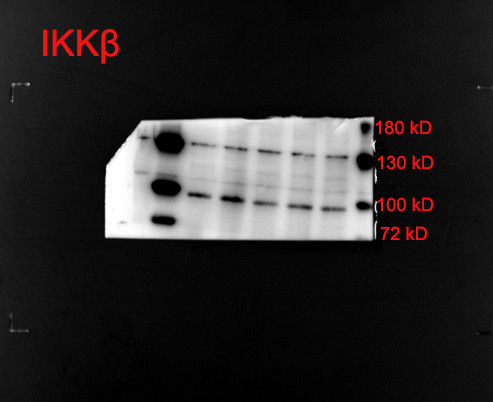


p-p65:


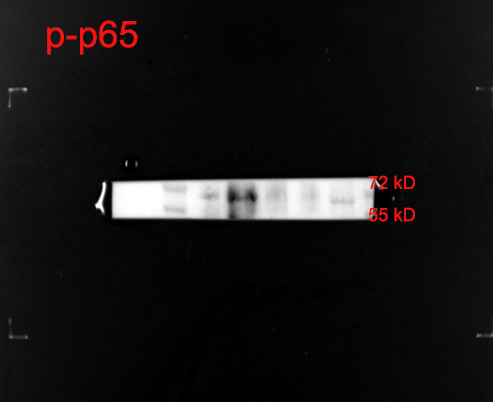

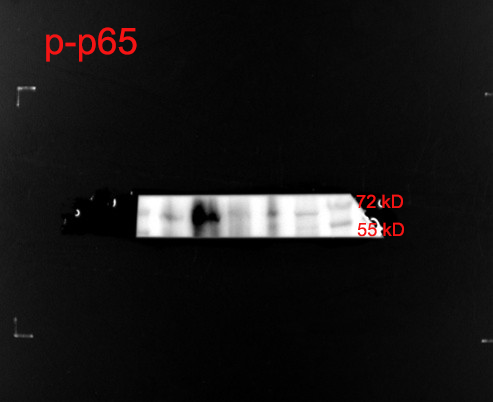

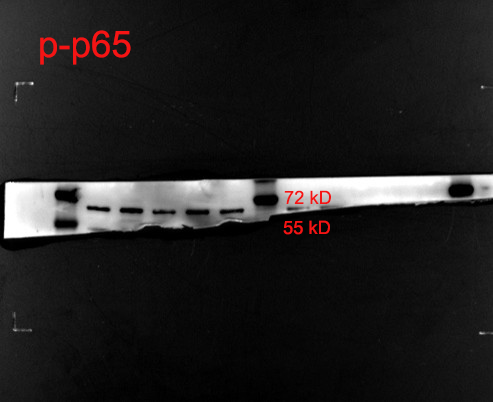


p65:


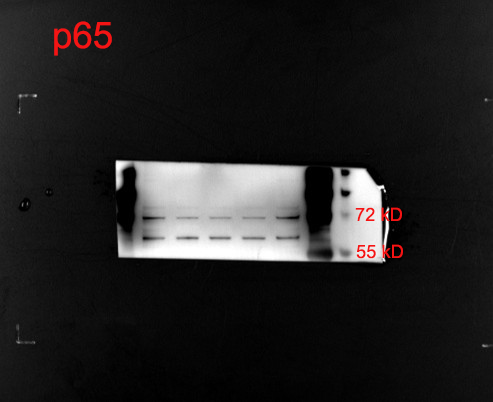

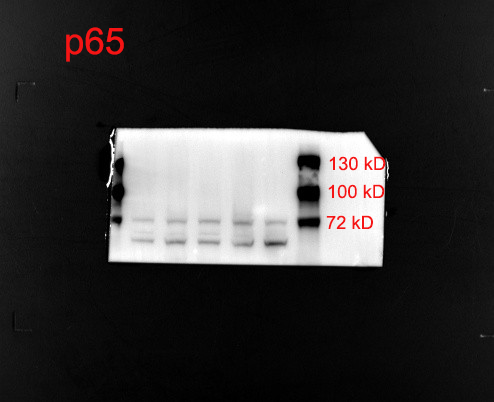

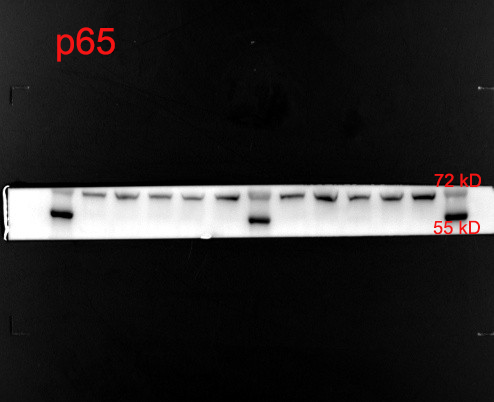


p-IκBα:


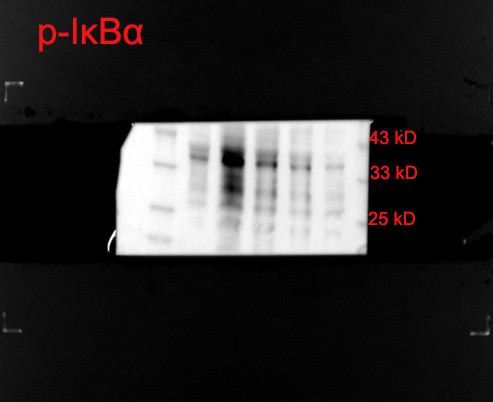

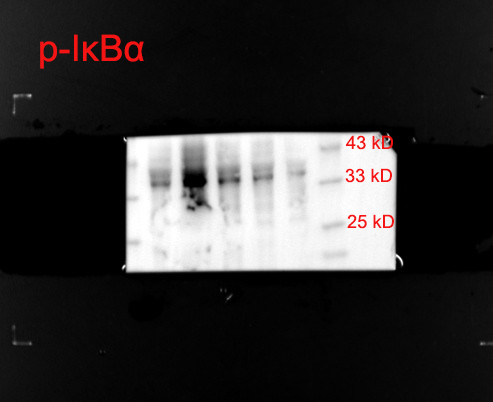

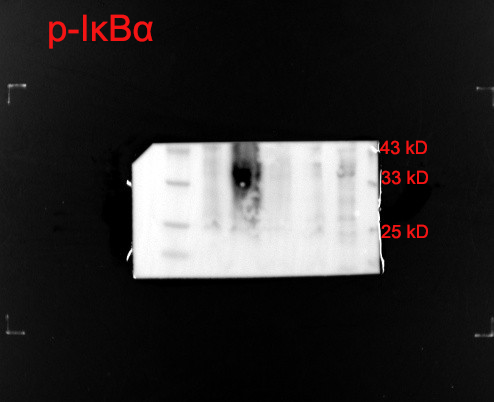


IκBα:


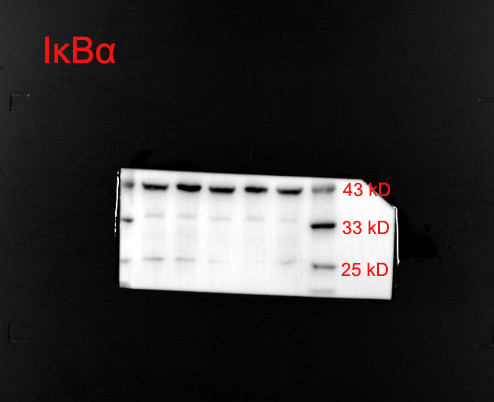

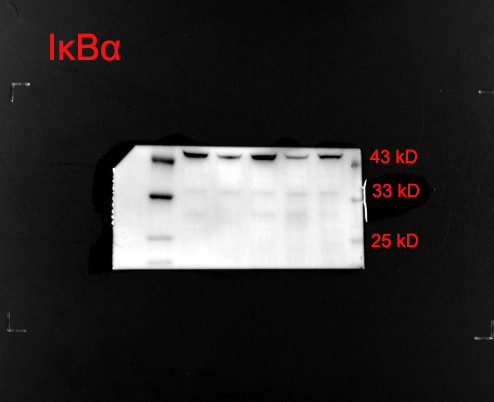

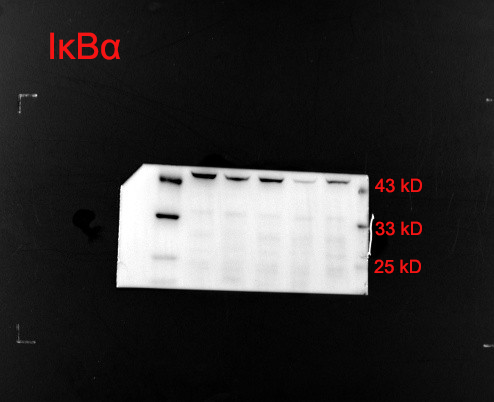


MyD88:


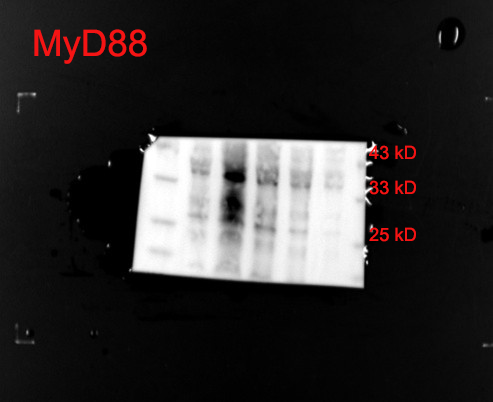

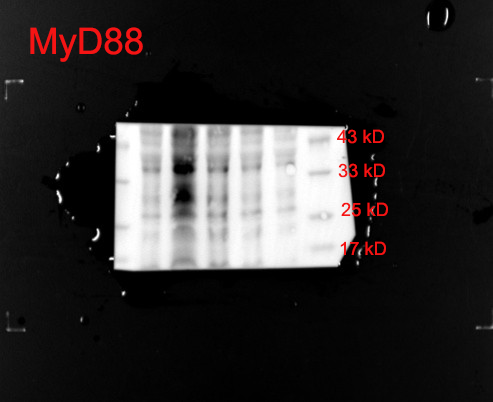

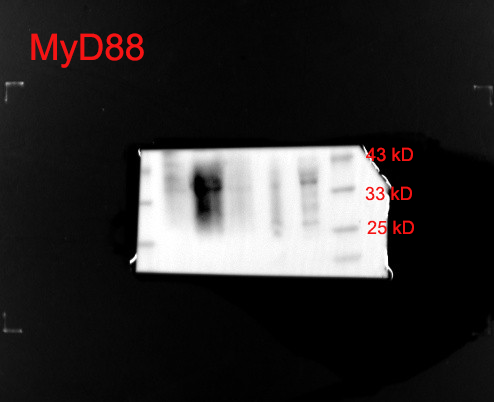


GAPDH:


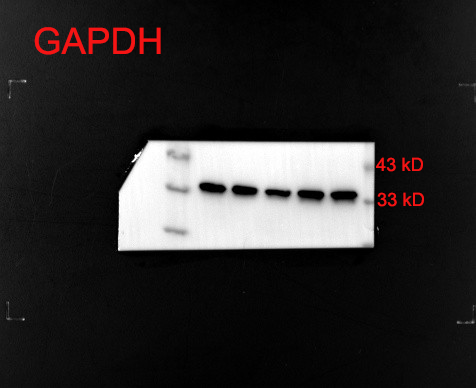

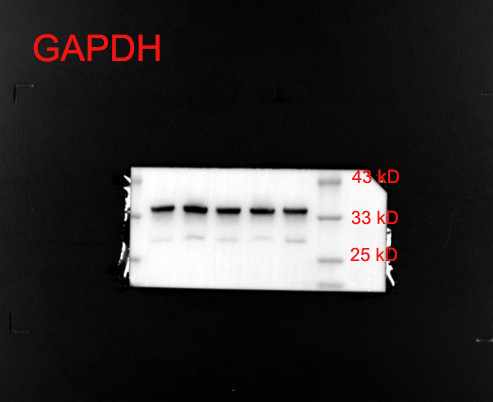

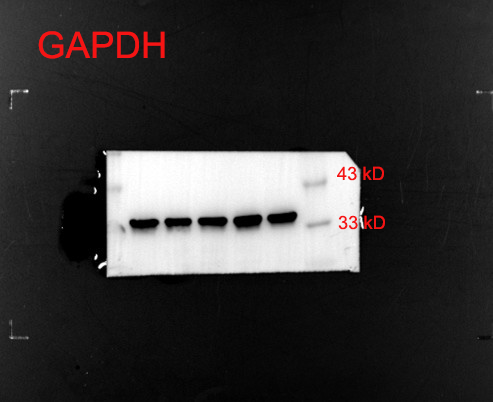


**Supplementary Fig. 3.** The original WB images of α-SMA, Collagen I, and proteins associated with the TLR4/NF-κB signaling pathway.

1. **Supplementary Tables**

**Supplementary Table S1.** **Gene primer sequences for real-time PCR.**

| **Gene name** | **Forward (5’-3’)** | **Reverse (5’-3’)** |
| --- | --- | --- |
| *α-SMA (human)* | *GTGTTGCCCCTGAAGAGCAT* | *GCTGGGACATTGAAAGTCTCA* |
| *Collagen I (human)* | *GAGGGCCAAGACGAAGACATC* | *CAGATCACGTCATCGCACAAC* |
| *GAPDH* *(human)* | *GGAGCGAGATCCCTCCAAAAT* | *GGCTGTTGTCATACTTCTCATGG* |
| *IL-1β* | *TCGCAGCAGCACATCAACAAGAG* | *AGGTCCACGGGAAAGACACAGG* |
| *IL-6* | *CTCCCAACAGACCTGTCTATAC* | *CCATTGCACAACTCTTTTCTCA* |
| *TNF-α* | *ATGTCTCAGCCTCTTCTCATTC* | *GCTTGTCACTCGAATTTTGAGA* |
| *TGF-β* | *CTTGCCCTCTACAACCAACA* | *ACTTGCGACCCACGTAGTAGA* |
| *GAPDH* | *GGTTGTCTCCTGCGACTTCA* | *TGGTCCAGGGTTTCTTACTCC* |

Molecular docking analysis revealed that 3-CP binds within the binding pocket of TLR4 (PDB ID: 3fxi) through hydrogen bonding interactions with residues ILE-114 and LEU-117, as well as hydrophobic contacts with ASN-143, ALA-139, and SER-140. For p65 (PDB ID: 1nfi), 3-CP formed hydrogen bonds with PRO-140 and GLN-142, while hydrophobic interactions were observed with LEU-175, and PRO-177. These interacting residues are located within functionally important regions of both proteins and have been previously implicated in ligand recognition.

**Supplementary Table S2. The binding energies and key residues for all ‎targets of TLR4/MYD88/NF-κB pathway.**

| **Targets** | | **PDB number** | **Binding energies**  **(kcal/mol)** | **Key residues** |
| --- | --- | --- | --- | --- |
| TLR4 | 3fxi | | -5.6 | ILE-114, LEU-117, ASN-143, ALA-139, SER-140 |
| MyD88 | 3mop | | -4.8 | ARG40, THR-48, GLU-52, TYR-58, GLU-65 |
| IκBα | 4kba | | -5.2 | ILE-23, LYS-38, GLU-52, MET-82, ILE-148, ASP-149 |
| IKKβ | 1r0p | | -5.4 | LYS-1110, LEU-1112, ILE-1115, LEU-1225, ARG-1227 |
| p65 | 1nfi | | -5.3 | PRO-140, GLN-142, LEU-175, PRO-177 |
| TRAF6 | 1lb4 | | -5.2 | VAL-74, CYS-99, GLU-100, GLY-102, ASP-103, ILE-165 |
| IRAK | 6bfn | | -5.6 | ILE-52, PHE-76, GLU-92, PHE-121, GLU-149 |
| TAK1 | 3p0u | | -6.1 | VAL-29, ARG-31, LEU-77, ALA-78 |

**Supplementary Table S3. Summarizes and compares the mechanisms of action, molecular targets, and clinical status of pirfenidone, obeticholic acid, and 3-CP.**

| **Compound** | | **Primary Mechanism** | **Molecular Targets** | **Clinical Status** |
| --- | --- | --- | --- | --- |
| **Pirfenidone** | Anti-fibrotic via TGF-β pathway inhibition; some antioxidant effects | | TGF-β, TNF-α, collagen synthesis | FDA-approved for idiopathic pulmonary fibrosis |
| **Obeticholic acid** | FXR agonist regulating bile acid metabolism and inflammation | | Farnesoid X receptor (FXR) | FDA-approved for primary biliary cholangitis; investigated for NASH |
| **3-CP** | Dual antioxidant and anti-inflammatory action | | ROS scavenging; TLR4/NF-κB pathway inhibition | Preclinical; investigated in liver fibrosis |
